# Discovery of Glycation-Derived Cross-links at Arginine

**DOI:** 10.1021/jacs.5c12902

**Published:** 2026-02-12

**Authors:** Jeremiah W. Jacob-Dolan, Amy C. Sterling, Morgan E. Brutus, Stefan M. Hansel, Rebecca A. Scheck

**Affiliations:** Department of Chemistry, 1810 Tufts University, 62 Talbot Ave, Medford, Massachusetts 02155, United States

## Abstract

Glycation cross-links account for more than 40% of all known advanced glycation end products (AGEs) and are correlated with many age-related diseases. Despite much interest, cross-linking AGEs (xl-AGEs) remain poorly understood, as they have been challenging to discover, prepare, and quantify. Here, we describe a peptide platform that is ideally suited for the study of xl-AGEs, which not only facilitates direct comparisons between the prevalence of known xl-AGEs and other AGEs but also enables the discovery of previously unknown xl-AGEs. In this study, we use this platform to discover the first known Arg–Arg xl-AGEs, a pair of **m** ethylglyoxal-derived dihydroxy **i** mi **d** azolidine hemi **a** cetal cross **l** ink, or MIDAL, isomers. We show that MIDAL can become the major AGE, exceeding levels of all other AGEs, for substrates in which two Arg glycation sites are optimally positioned. We further demonstrate that MIDAL is readily and reversibly generated in biocompatible conditions, persisting with a half-life of more than 3 days. We also demonstrate that MIDAL can form in living mammalian cells, suggesting that it has the potential to be a dynamic, physiologically relevant and functional xl-AGE. This work therefore offers important insights about MIDAL formation and describes a versatile platform to enable the study of xl-AGEs under a variety of conditions. We expect that it will be highly useful for further discovery of biologically relevant glycation cross-links that are yet to be identified.

## Graphical abstract

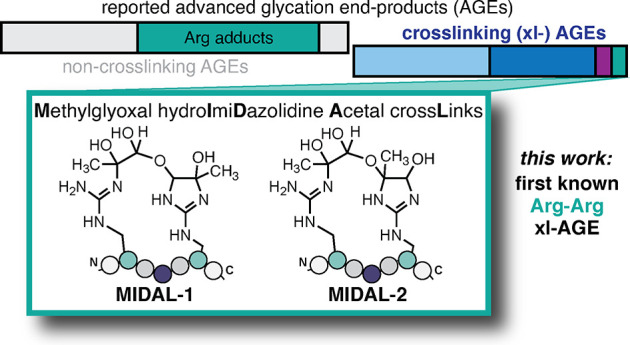

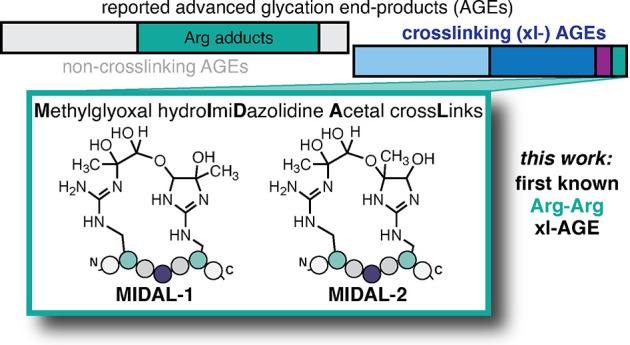

## Introduction

Covalent protein cross-links are involved in an impressive range of cellular functions, ranging from strengthening extracellular matrices,^1–7^ assisting in bacterial invasion,^8^ regulating the cytoskeleton,^9^ participating in signaling cascades,^10–12^ or stabilizing intramolecular protein folds.^13–15^ While some covalent cross-links are installed or removed by enzymes, others transpire nonenzymatically, often due to oxidative or metabolic stress.^16,17^ For example, cross-linking advanced glycation end products (AGEs) are thought to be a major source of age-related protein damage, especially for long-lived proteins of the extracellular matrix.^18,19^ These cross-linking AGEs form spontaneously from endogenous sugars and sugar-derived metabolites, producing a range of harmful impacts including weakened bone strength,^20^ increased myocardial stiffness,^21^ and the formation of cataracts^22^ or age-related macular degeneration.^23^ As the majority of cross-linking AGEs are associated with aging, they are typically thought to accumulate slowly over time.^18,19^ However, recent work has shown that some AGE cross-links form rapidly, even playing a functional role in activation of a bacterial phospholipase^24^ or the antioxidant response.^25,26^ Together, these examples suggest that xl-AGEs perform a range of important functions in cellular signaling and human health. However, current efforts to study cross-linking AGEs have so far been unable to facilitate a clear understanding of their prevalence and are poorly suited for discovering new ones.^27^

AGEs are a chemically heterogeneous set of protein post-translational modifications (PTMs) that are formed through the nonenzymatic browning process known as glycation, in which aldehydes or ketones become covalently linked to biomolecules (Figure 1).^28–32^ While 43% of reported AGEs are cross-links, the rest are individual AGEs that modify just a single Arg, Lys, Cys, or N-terminus (Figure 1A), such as the methylglyoxal-derived hydroimidazolone (MGH-1) and carboxymethyllysine (CML) isomers.^30,33^ Notably, Arg is a major site of glycation, especially by biologically relevant dicarbonyls such as methylglyoxal (MGO) (Figure 1B).^34^ Despite the importance of Arg as a glycation site, to date, there have been no xl-AGEs reported between two Arg residues. Instead, any known Arg-containing xl-AGEs also include other side chains such as Lys or Cys (Figure 1C).^26,28,35,36^ Additionally, compared to non-cross-linking (or mono-) AGEs, relatively little is known about cross-linking (xl-) AGEs. For example, while glucosepane is thought to be a major xl-AGE,^32,37^ we have been unable to find any studies that quantify its prevalence relative to common AGEs such as the MGH isomers or CML.

**Figure 1:**
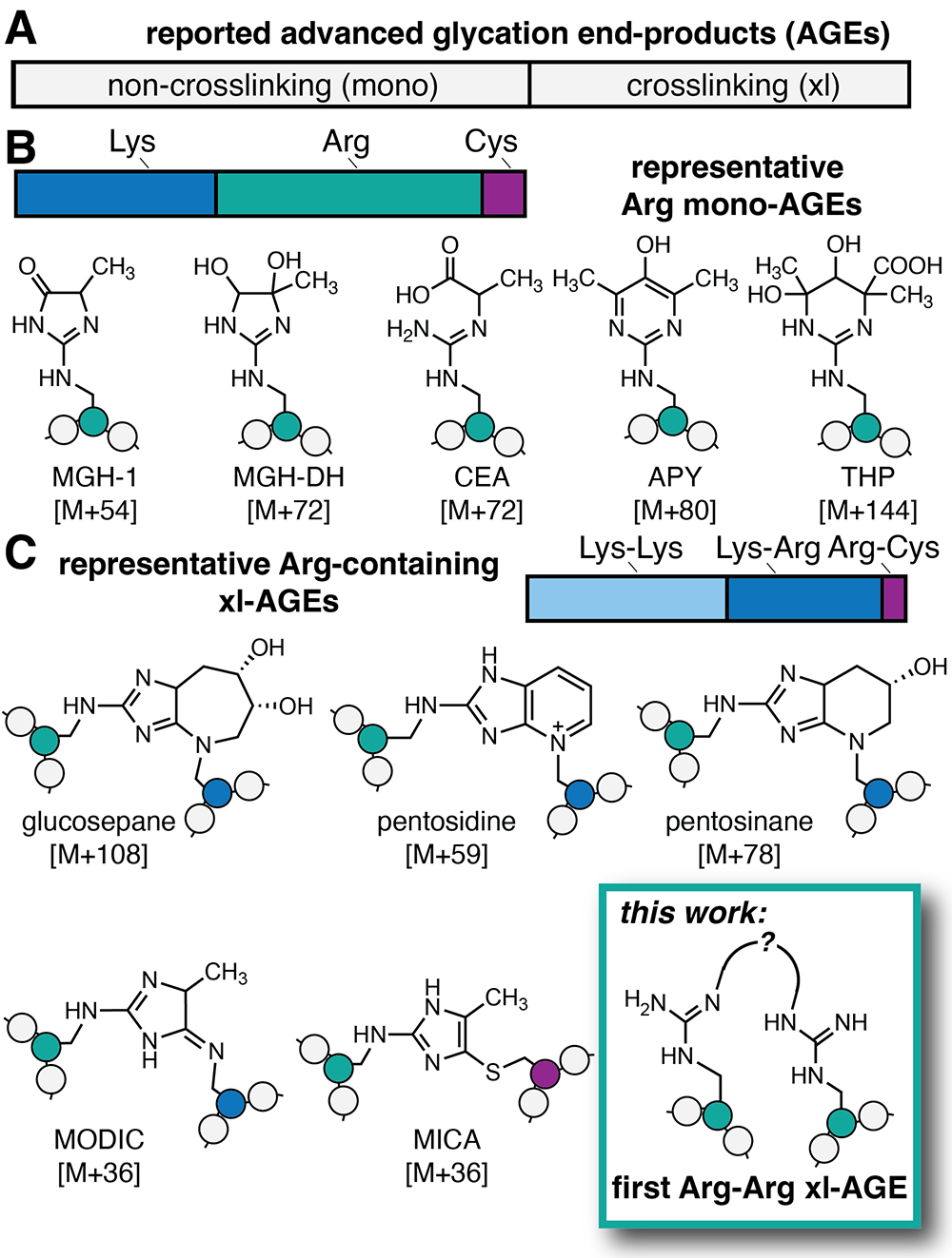
(A) Of the 40 known advanced glycation end products (AGEs) that have been reported, almost half (17) are cross-linking AGEs. (B) For non-cross-linking (referred to as “mono-”) AGEs, the vast majority are found at Lys (9) and Arg (12). Arg is preferentially glycated by the biologically relevant glycating agent methylglyoxal (MGO), forming several AGEs including the methylglyoxal-derived hydroimidazolone isomers (*e.g*., MGH-1, shown), dihydroxyimidazolidine (MGH-DH), carboxyethylarginine (CEA), argpyrimidine (APY), and tetrahydropyrimidine (THP). (C) Known cross-linking (xl-)­AGEs are formed mostly between two Lys (9) or a Lys-Arg residue (7), but prior to this work, none have been reported between two Arg. Representative Arg-containing xl-AGEs include glucosepane, pentosidine, pentosinane, methylglyoxal-derived imidazolium cross-link (MODIC), and mercaptomethylimidazole cross-links between cysteine and arginine (MICA). Here, we report the first Arg–Arg MGO-derived cross-link, which we have named MIDAL.

In this work, we set out to develop a peptide-based discovery platform that would enable the study of glycation cross-links. Here, we show that the platform we built not only is suitable for making direct comparisons between known xl-AGEs and mono-AGEs but also enables the discovery of previously unknown xl-AGEs. Specifically, herein, we describe the first reported Arg-Arg xl-AGEs, a pair of **m** ethylglyoxal-derived dihydroxy **i** mi **d** azolidine hemi **a** cetal cross **l** ink isomers, which we have named MIDAL. For substrates with optimally positioned Arg, MIDAL becomes the major AGE, surpassing even the formation of non-cross-linking AGEs. We further demonstrate that MIDAL is generated on short time frames and persists for days under mild, biocompatible conditions. Finally, we show that MIDAL can form in living mammalian cells, suggesting that it has the potential to be a major contributor to the glycation landscape. Our findings suggest not only that MIDAL could be a functional xl-AGE but also that there may be additional biologically relevant glycation cross-links that are yet to be identified.

## Results

Our lab has previously shown that synthetic peptides are useful substrates for evaluating glycation chemistry.^34,38^ In this work, we sought to develop a peptide-based platform that would be particularly well-suited to identify AGE cross-links, especially those that are Arg-derived. To do so, we envisioned that it would be possible to discern xl-AGEs from any other AGEs by placing a protease recognition site in between glycation sites. Upon glycation, a mixture of mono-AGEs and xl-AGEs would be obtained. However, formation of xl-AGEs would render the sequence recalcitrant to enzymatic cleavage. Therefore, any AGEs remaining on full-length peptides after digestion would indicate that a cross-link had formed (Figure 2A). We opted to incorporate Gly and Pro as intervening residues, encouraging conformations in which the two Arg face each other and are primed for cross-link formation (Figure S1). We therefore initiated this study by synthesizing a small library of peptides (peptides **1**–**5**) in which two Arg were separated by a single Pro and a variable number of Gly spacers (Table S1). To avoid any potential for glycation at the N-terminus, peptides were acetylated.

**Figure 2:**
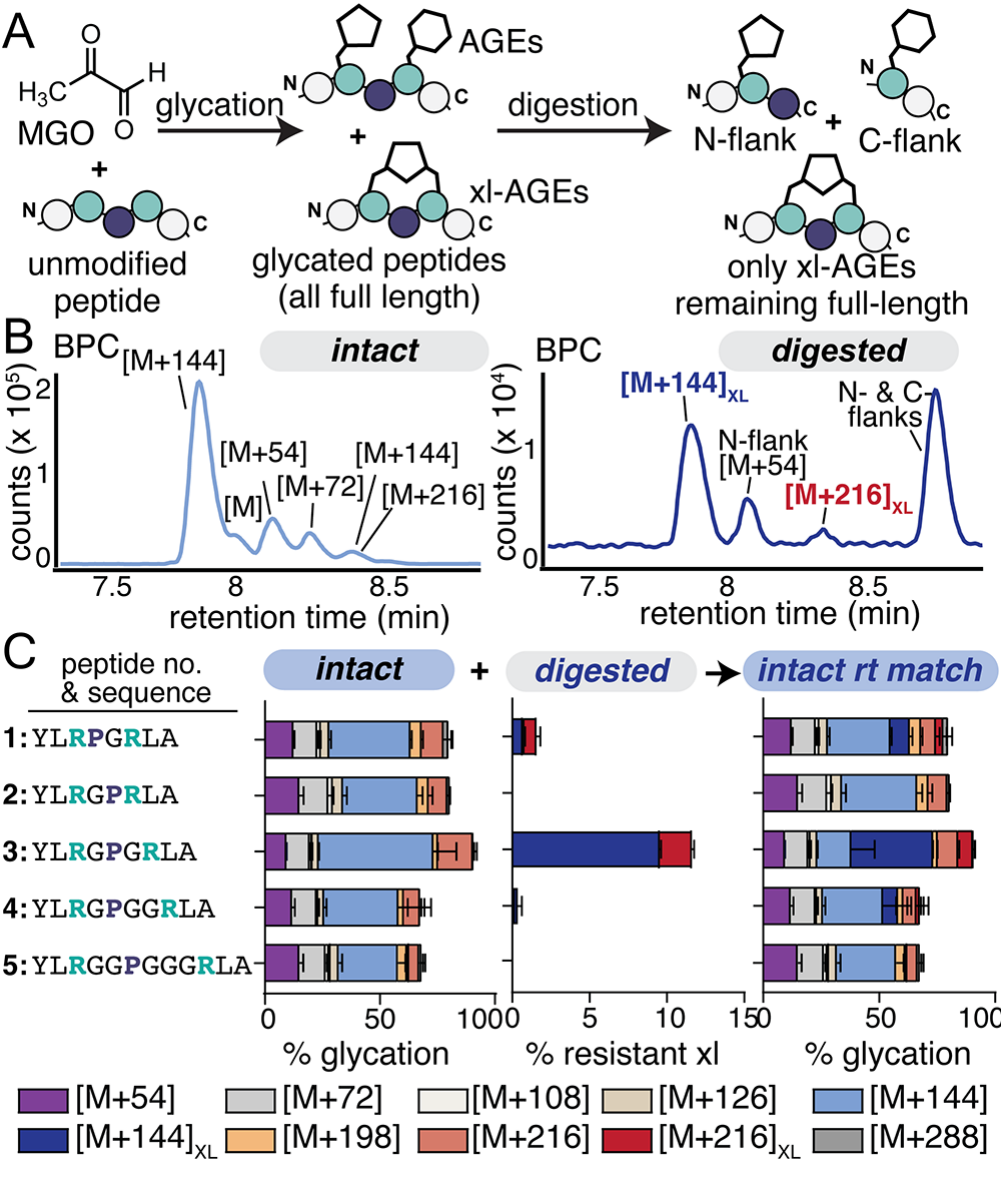
(A) To evaluate the formation of Arg–Arg glycation cross-links derived from MGO, we developed a peptide platform in which xl-AGEs could be discerned from all others after cleavage of the peptide backbone by a Pro-specific peptidase. Scheme depicting the use of this platform to screen a small library of peptides (peptides **1**–**5**) in which two Arg (green) were separated by Pro (navy)–Gly linkers of varying lengths. Peptides (1 mM) were treated with 2 mM MGO for 24 h at 37 °C in 2× phosphate-buffered saline (PBS) at pH 7.4. After quenching the glycation reaction with Tris buffer (intact), a portion of the sample was treated (digested) with the Pro peptidase prior to analysis by liquid chromatography mass spectrometry (LC-MS). (B) Representative base peak chromatogram (BPC) for MGO-glycated peptide **3** showing intact (*left*) and digested (*right*) samples. (C) Distribution of AGE adducts observed via LC-MS for peptides **1**–**5** on intact samples (*left*) and on remaining full-length peptides after digestion (*middle*). By matching retention times, intact data was reanalyzed to differentiate xl-AGEs from other AGEs (*right*). Stacked bar graphs are shown with mean ± standard deviation for each adduct. Data is from independent experiments, with *n* = 9 for peptide **3** and *n* = 3 for all other peptides. Legend: purple [M+54], light gray [M+72], cream [M+108], tan [M+126], light blue [M+144], dark blue [M+144]_XL_, orange [M+198], light red [M+216], dark red [M+216]_XL_, and dark gray [M+288].

Crucially, the Pro-containing intervening sequence is also a recognition sequence for a Pro-specific endopeptidase.^39^ We also optimized proteolysis conditions and found that the Pro peptidase exhibited highly efficient cleavage on these short peptide substrates, as compared to tobacco etch virus (TEV) on a related set of peptides (Figure S2). We confirmed that unmodified peptide was completely cleaved by the Pro peptidase after only 15 min at 30 **°**C. However, we note that the Pro peptidase exhibited modest off-target cleavage for sequences in which an aromatic residue was immediately C-terminal to Arg (Figure S3). This behavior guided our design, as we avoided placing Tyr immediately before or after either of the Arg sites.

Next, we evaluated the use of this platform to reveal xl-AGEs by treating each peptide (**1**–**5**, 1 mM) with 2 mM MGO for 24 h at 37 **°**C (Figure 2A–C). Although outside of a physiological range, these conditions are similar to those we have used to assess peptide glycation in the past,^38^ though the MGO concentration was doubled to account for the additional Arg residue. After quenching the glycation reaction in Tris buffer, a portion of the sample was further treated with the Pro peptidase prior to analysis by liquid chromatography–mass spectrometry (LC-MS). Using this approach, we observed the expected Arg AGE adducts, including MGH-1 ([M+54]), CEA and MGH-DH ([M+72]), and multiple [M+144] adducts, which likely include THP (Figure 2B, *left*.) As there were two potential glycation sites in our substrate, we also observed expected mass changes that aligned with combinations of these adducts (e.g., double MGH formation, [M+108]), up to [M+288]. These were observed in roughly similar distributions for all peptides tested (Figure 2C, *left*).

By contrast, after digestion, no unmodified peptide remained, and many of these high molecular weight AGEs were no longer present (Table S2). Instead, we observed the unmodified N- and C-terminal flanks (*m*/*z* = 324.1800, *z* = 2 (observed); 324.1739, *z* = 2 (expected) and 208.5792, *z* = 2 (observed); 208.6344, *z* = 2 (expected), respectively), liberated after digestion, as well as glycated versions of these fragments (Figure 2B, *right*). Of particular interest, however, were two species (*m*/*z* = 594.8244 and 630.8343, respectively (*z* = 2)), both of which were greater than the mass of the unmodified parent peptide we began with (*m*/*z* = 522.8034, *z* = 2). These results suggested that our platform is successfully able to differentiate xl-AGEs from others that form (Figure 2C, *middle*).

Although we had predicted that only xl-AGEs would be remaining as full-length (or greater) masses after digestion, we also envisioned two possibilities for this behavior. One possibility was that xl-AGEs would be inert to the Pro peptidase; in this case, we expected to see identical retention times in both intact and digested samples. The other possibility was that the backbone would still be clipped during digestion, leaving a full-length xl-AGE peptide with the addition of water. Careful inspection of retention times (r.t.) and mass changes observed in both intact and digested samples revealed that the observed xl-AGEs were identical in both treatments. Specifically, one xl-AGE adduct ([M+144]_XL_) eluted at r.t. = 7.855 ± 0.012 min with *m*/*z* = 594.8244, *z* = 2 in both intact and digested samples. The other xl-AGE ([M+216]_XL_) eluted as a mixture of isomers with retention times at 8.180 ± 0.009 and 8.381 ± 0.004 min, both with *m*/*z* = 630.8343, *z* = 2 that were identical in digested samples. These results suggest that xl-AGEs are indeed resistant to Pro peptidase cleavage, consistent with past studies evaluating proteolysis of cyclic or other constrained peptides.^40^ As it was difficult to quantify the extent of cross-link formation due to digestion of unmodified parent peptide and an apparent loss of counts after digestion (Figure S3), we instead estimated it as a “% resistant cross link” (Figure 2C, *middle*) and used the digestion protocol solely to determine which AGEs were cross-links.

Having used the digestion protocol to determine the exact retention time for each of the two xl-AGEs in the intact samples, it was possible to perform superior quantification that enabled us to directly compare the relative levels of xl-AGEs to mono-AGEs (Figure 2C, *right*). We found that only peptide **3** (Ac-YLRGPGRLA) led to substantial levels of xl-AGE formation, with 40.2 ± 4.8% of [M+144]_XL_ and 7.1 ± 0.9% of [M+216]_XL_. Peptide **3** generated 5-fold the levels of xl-AGE formation compared to peptide **1** (8.4 ± 1.2%), and peptide **4** generated even less (6.4 ± 5.9%). Additionally, no xl-AGEs were observed for peptides **2** or **5**, suggesting that the distance between the two Arg residues is likely to play a major role in the amount of cross-link formed (Figure 2C). Moreover, [M+144]_XL_ was the highest abundance adduct out of all AGEs observed for peptide **3.**

To ensure that the [M+144]_XL_ cross-link was not an artifact of the Pro peptidase, we also synthesized a peptide **3** variant that could be digested using a photolabile linker rather than enzymatic cleavage (Figure S4). After photocleavage, in addition to the single AGEs formed on the photocleaved fragments, we observed that only two masses remained at or above that of the parent peptide, which matched with the [M+144]_XL_ and [M+216]_XL_ adducts that we found using the Pro peptidase protocol. Taken together, these experiments confirm that the [M+144]_XL_ adduct is a previously unknown MGO-derived cross-link that forms between two Arg. We further suspect that the [M+216]_XL_ is an additional MGO addition (either MGH-DH or CEA) on peptides already containing [M+144]_XL_, as has been previously suggested for related xl-AGEs.^41^

While there have been no other reported Arg-Arg AGE cross-links, and no xl-AGEs reported with mass changes of [M+144] or [M+216], there are other known MGO-derived cross-links that include Arg at one of the reactive sites. To further confirm that we were observing authentic cross-link formation, we therefore sought to use our peptide platform to confirm the presence of known cross-links. In particular, we focused on two known MGO-derived cross-links: an Arg – Cys cross-link (MICA) and an Arg–Lys cross-link (MODIC) (Figure 3A). To do so, we prepared peptide substrates that resembled peptide **3** but included a single-point mutation changing the Arg at position 3 to a cysteine (peptide **6**) or a lysine (peptide **7**).

**Figure 3:**
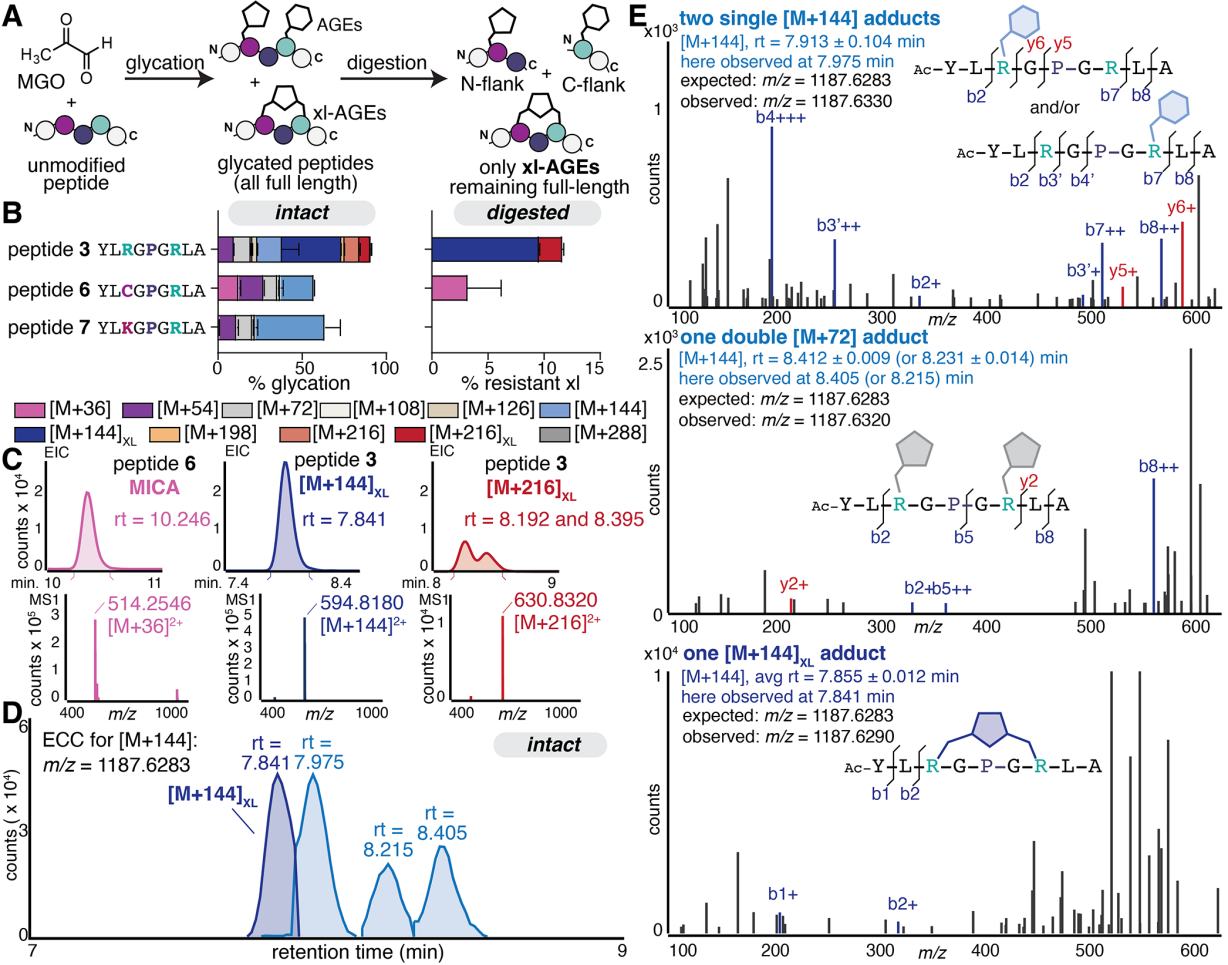
(A) To confirm cross-link formation, variations of peptide **3** were prepared with Cys (peptide **6**) or Lys (peptide **7**), which are known to form the cross-links MICA or MODIC, respectively, when treated with MGO. Scheme depicting the treatment of peptides (1 mM) with 2 mM MGO for 24 h at 37**°**C in 2× phosphate-buffered saline (PBS) at pH 7.4. After quenching the glycation reaction with Tris buffer (intact), a portion of the sample was treated (digested) with the Pro peptidase prior to analysis by LC-MS. (B) Distributions of AGE adducts observed via LC-MS on peptides **3**, **5**, and **6** for intact samples (*left*) and on remaining full-length peptides after digestion (*right*). By matching retention times, intact data was reanalyzed to differentiate xl-AGEs, as shown in the intact AGE distributions (*left*). Stacked bar graphs are shown as the mean ± standard deviation for each adduct. Data is from independent experiments, with *n* = 9 for peptide **3** and *n* = 3 for all other peptides. Legend: pink [M+36], purple [M+54], light gray [M+72], cream [M+108], tan [M+126], light blue [M+144], dark blue [M+144]_XL_, orange [M+198], light red [M+216], dark red [M+216]_XL_, and dark gray [M+288]. (C) Representative extracted ion chromatograms (EIC) for cross-linking adducts and corresponding mass spectra. (D) Representative extracted compound chromatograms for four discrete [M+144] isomers from the same intact sample of peptide **3** treated with MGO. (E) MS^2^ spectra for [M+144] isomers (precursor ion *m*/*z* = 1187.6283). Diagnostic b and y ions were found for the isomer at observed at retention times of 7.975 min (b2, b3′, b4’, y6, y5, b7, and b8), suggesting a single THP (or other [M+144] mono-AGE) (*top*), and for those at 8.405 and 8.215 min (b2, b5, and y2) suggesting two [M+72] mono-AGEs, one on each Arg (*middle*). For the [M+144]_XL_ isomer observed at a retention time of 7.841 min, no diagnostic b and y ions were identified, providing further evidence of cross-link formation (*bottom*).

Unlike the xl-AGEs we identified for Arg-only sequences, both MICA and MODIC cross-links have a unique mass change ([M+36]) that is conveniently tracked even without digestion. When treated with MGO, we found that both peptides **6** and **7** were highly AGE modified (57.5 ± 2.3 and 63.4 ± 9.9% respectively), though peptide **3** produced the highest level of overall glycation (90.6 ± 8.1%). Prior to digestion, we observed an [M+36] mass adduct for peptide **6** (12.6 ± 1.0%) suggesting that MICA is able to form under the conditions used in this study. For peptide **7**, an [M+36] adduct was observed in vanishing quantities (0.1 ± 0.2%) but only in intact samples, suggesting minimal MODIC formation that was not sufficient to survive digestion (Figure 3B). This difference may be attributed to the fact that MGO reacts quickly with both Arg and Cys but reacts far more slowly with the Lys ε-amine.^42^ It could also be due to a structural effect that causes peptide **7** to adopt conformations in which side chains are not optimally placed for cross-linking.

Representative extracted ion chromatograms (EIC) for all cross-links observed show that MICA and [M+144]_XL_ elute as single, well-resolved peaks, but [M+216]_XL_ appears as multiple, broad peaks and in far lower quantities (7.1 ± 0.9%) of total peptide volume compared to [M+144]_XL_ (40.2 ± 4.8%) (Figure 3C). For these reasons, we focused our attention on [M+144]_XL_ and its comparison to MICA. Specifically, the discrete mass change for MICA allowed us to directly compare levels of [M+36] in both intact and digested samples. We found that MICA (r.t. = 10.260 ± 0.009 min; *m*/*z* = 514.2574, z = 2) was the only AGE to survive digestion for peptide **6** (Figure 3B). After digestion, there was a substantial loss of absolute counts, which were decreased by an order of magnitude (Figure S5). This matches the behavior we observed for [M+144]_XL_ on peptide **3**. We suspect that the loss of counts after digestion could be due to either off-target cleavage from the peptidase or on-target cleavage that destabilizes the cross-link. Nonetheless, these data support the use of the Pro peptidase to confirm the exact mass and retention time of each xl-AGE, with any quantification relative to other AGEs taking place on the intact (undigested) sample. By doing so, we found that the [M+144]_XL_ we identified appears to be of greater prevalence than MICA for comparable substrates (40.2 ± 4.8 vs 12.6 ± 1.0%, respectively) (Figure 3B,C). Unless otherwise noted, all subsequent AGE distributions reported are based on quantification from intact samples, with retention time matching between intact and digested samples performed in a pairwise manner.

Unlike MICA and MODIC, the new cross-link that we uncovered does not have a mass that can be easily differentiated from other AGEs. With two Arg, there are multiple AGE combinations that would each produce an identical mass change of [M+144], including the formation of tetrahydropyrimidine (THP), other AGEs with a mass change of [M+144], or the formation of [M+72] (MGH-DH or CEA) on both Arg sites. Accordingly, we were able to observe four [M+144] adducts with discrete retention times during our intact experiment (Table S2 and Figure 3D). As further confirmation of cross-link formation, we performed targeted tandem mass spectrometry (MS^2^) analysis on [M+144] species (precursor ion *m/z* = 594.8, isolation width = 4 *m*/*z*). For the peak eluting at 7.913 ± 0.104 min (observed in a representative sample at 7.975 min), the resulting MS^2^ spectra were consistent with the formation of a single THP (or other [M+144] mono-AGE) on either Arg, with both species coeluting. Diagnostic ions included b2 and y6 ions, showing modification of the first Arg (at position 3), and the b4′ and b7′ ions showing modification of the second one (at position 7) (Figure 3E, *top*). At a retention time of 8.412 ± 0.009 (Figure 3E, *middle*) and 8.231 ± 0.014 min (observed at 8.405 and 8.215 min, respectively), the b2, b5, and y2 ions are diagnostic for another [M+144] species that has two [M+72] modifications, one at each Arg. Finally, in the MS^2^ analysis for [M+144]_XL_ at 7.855 ± 0.012 min (Figure 3E, *bottom*) (observed at 7.841 min), no diagnostic ions were observed. The only b or y ions that could be assigned were for b1 or b2, which fall outside of the cross-link itself. We expect that this occurs because MIDAL forms a cyclic peptide that prevents the expected fragmentation that would usually result from collision-induced fragmentation at amide bonds during conventional MS^2^. While some known intermolecular cross-links between linear peptides^43,44^ or C- to N-cyclized peptides can be sequenced by MS^2^ with nonconventional fingerprints,^45,46^ we have not found any examples of MS^2^ analysis in which a nonamide cross-link is responsible for cyclization. Thus, we interpret the inability to identify any diagnostic ions as further evidence of cross-link formation.

Although many AGE cross-links have been reported, none have a mass change of [M+144], suggesting that the one we discovered has a novel structure. To aid in determining the [M+144]_XL_ structure, we used two MGO derivatives (Figure 4A). First, we used a deuterated version of MGO (D4-MGO), in which all four protons are replaced with deuterium. Experiments using deuterated MGO exhibited no exchange of protons during cross-link formation, generating an [M+152]_XL_ adduct that differed by the expected 8.00 Da from [M+144]_XL_. We also used glyoxal (GO) to show that a methyl group was not required to form the cross-link, as we observed an [M+116]_XL_ adduct that corresponds to the [M+144]_XL_ obtained for MGO (Figure 4B,C).

**Figure 4:**
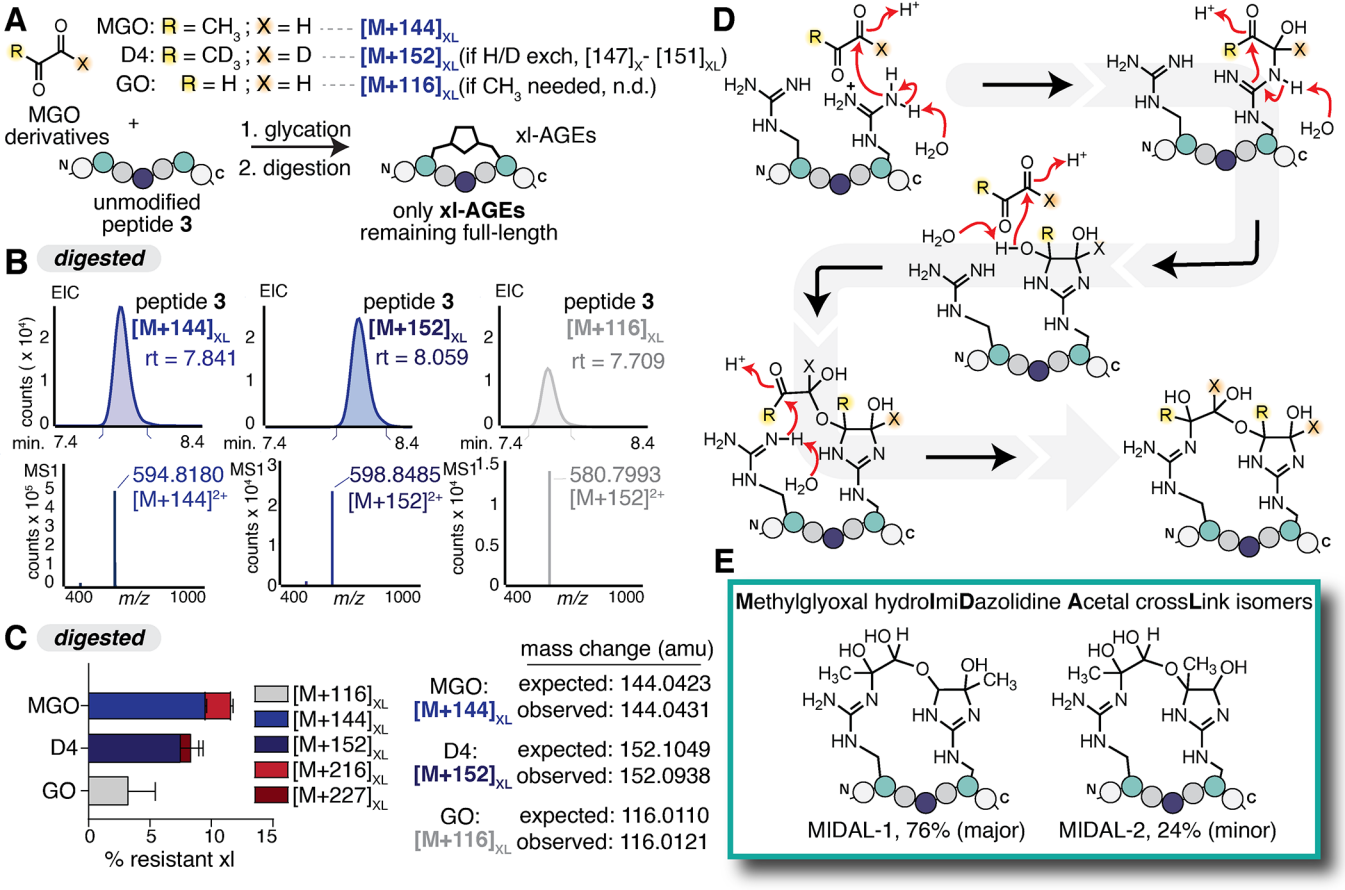
(A) Glycation reactions were performed by incubating 1 mM peptide **3** with either 2 mM MGO, 2 mM deuterated (D4)-MGO, or 5 mM glyoxal (GO) in 2× PBS at pH 7.4 for 24 h at 37 °C, followed by digestion with the Pro peptidase to track formation of xl-AGEs. (B) Representative extracted ion chromatograms (EIC) and corresponding MS spectra for xl-AGEs remaining at or above the parent mass after glycation and subsequent digestion. (C) AGE distributions for digested samples after glycation. Expected and observed mass changes for each MGO derivative are shown. Stacked bar graphs show the mean ± standard deviation for each xl-AGE adduct, quantified on the digested samples. Data is from independent experiments, with *n* = 9 for peptide **3** treated with MGO and *n* = 3 for D4-MGO and GO treatments. Legend: light gray [M+116]_XL_, blue [M+144]_XL_, dark blue [M+152]_XL_, red [M+216]_XL_, and dark red [M+227]_XL_. (D) Proposed mechanism for [M+144]_XL_ formation that is consistent with observations in (B) and (C). (E) NMR characterization supports the assignment of methylglyoxal-derived dihydroxyimidazolidine hemiacetal crosslink (MIDAL) isomers (MIDAL-1, 76%; MIDAL-2, 24%). Structural characterization can be found in Supplementary Figures S6–S9.

While many reported AGEs suggest involvement of the methyl group and/or proton exchange, prior work outside of the glycation literature has suggested that dihydroxyimidazolidines can condense with an additional carbonyl, forming an acetal.^47,48^ Though past work has focused on this kind of linkage as a stable, standalone modification, we envisioned that similar chemistry could be at play in generating a cross-link. However, acetal formation between two MGO [M+126] would be inconsistent with the observed mass change. As a result, we instead considered structures and mechanisms in which no waters were lost. This led us to consider an alternative pathway where the nearby Arg catches and thereby stabilizes the hemiacetal intermediate. Such a mechanism would be consistent with our findings when using the MGO derivatives, as all MGO protons and the methyl group do not participate in cross-link formation (Figure 4D). Finally, using a combination of 1D and 2D NMR, we were able to confirm the structure of the [M+144]_XL_ cross-link, which involves a **m** ethylglyoxal-derived dihydroxy **i** mi **d** azolidine hemi **a** cetal cross **l** ink between Arg, which we have named MIDAL (Figure 4E and Figures S6–S9).

Having confirmed the structure of MIDAL, we next sought to determine if it could be a major AGE for other peptides containing the central RGPGR motif we identified as optimal for peptide **3**. To do so, we conducted a BLAST query against -RGPGR- to identify natural sequences that might also be able to form MIDAL. This search resulted in only four hits with 100% sequence overlap. Three were found in human immunoglobulin heavy chain junction regions, and one was from the HIV-1 P protein. We chose to synthesize three short peptide mimetics based on two of the immunoglobulin hits and the HIV-1 P hit. These sequences (peptides **8**–**10**) were subsequently evaluated for their ability to form MIDAL (Figure S10). After incubation with MGO for 24 h, we found that all three peptides formed MIDAL. While peptide **3** still exhibited the greatest amount of MIDAL formation (and overall glycation), peptide **9** also produced MIDAL as its major AGE (19.8 ± 3.3%). Additionally, while peptides **8** and **10** formed less MIDAL (10.3 ± 6.1 and 5.6 ± 1.8%, respectively), it was found in roughly equal proportion to other AGEs. Together, these results suggest that MIDAL prevalence rivals that of other common mono-AGEs and has the potential to become the major AGE on multiple substrates.

Next, to provide insight into the potential for MIDAL to form in cells, we evaluated the conditions that promoted its formation. We subjected peptide **3** to a variety of different glycation reaction conditions, scanning reaction time, temperature, MGO concentration, and pH (Figure S11). These data showed that MIDAL formation was maximal at neutral pH (7.4) and 37 **°**C. Furthermore, our results suggest that MIDAL is likely to form quickly, as it reaches its maximal levels in our *in vitro* system around 24 h (40.2 ± 4.8%), before it begins rearrangement into mono-AGEs such as MGH-DH and CEA. After 24 h of additional MGO incubation, MIDAL levels drop to 20.3 ± 3.1%. To determine the stability of MIDAL in the absence of MGO, we incubated purified MIDAL-modified peptide **3** at 37 °C in PBS at pH 7.4. Under these conditions, 43.7 ± 3.0% of MIDAL survived after a 4-day incubation (Figure S12). The removal of MGO from further incubation showed a modest increase in the stability of MIDAL, revealing its potential to be modulated by the available level of glycating agent (in this case MGO). Using these conditions, we calculated the half-life of MIDAL to be 3.3 days, which is longer than early AGEs such as MGH-DH, (*t*_1/2_ = 1.8 days)^49^ but shorter than AGEs such as fructosyl-lysine (*t*_1/2_ = 25 days) or the MGH isomers (*t*_1/2_ = 12 days).^50,51^ Collectively, these data demonstrate that MIDAL fits the profile of a dynamic and biologically relevant AGE, which not only forms readily but also persists for several days at physiological pH and temperatures.

Having both confirmed the structure of MIDAL and established that it forms under mild conditions, we sought to determine if it had the potential to form in a cellular environment. To do so, we transiently transfected HEK-293T cells with a plasmid in which the peptide **3** (-YLRGPGRLA, GFP-**3**), peptide **6** (-YLCGPGRLA, GFP-**6**), or peptide **7** sequence (-YLKGPGRLA, GFP**-7**) was fused at the C-terminus of green fluorescent protein (GFP) (Figure 5A). In addition to the internal Pro peptidase recognition site, these C-terminal sequences were connected to GFP through a linker sequence containing a tobacco etch virus (TEV) protease cleavage site (Figure 5B). We confirmed that all three variants were comparably expressed in HEK-293T cells after 24 h of transient transfection (Figure 5C). Next, to observe cross-link formation, we incubated transfected cells with or without 10 mM MGO for 2 h (*Treatment A*, Figure 5A). These treatment conditions were used to maximize cross-link formation while also minimizing MGO toxicity. Specifically, we found that even after 2 h of incubation with these high concentrations of MGO, >80% of cells remained viable, consistent with previous findings.^52^

**Figure 5:**
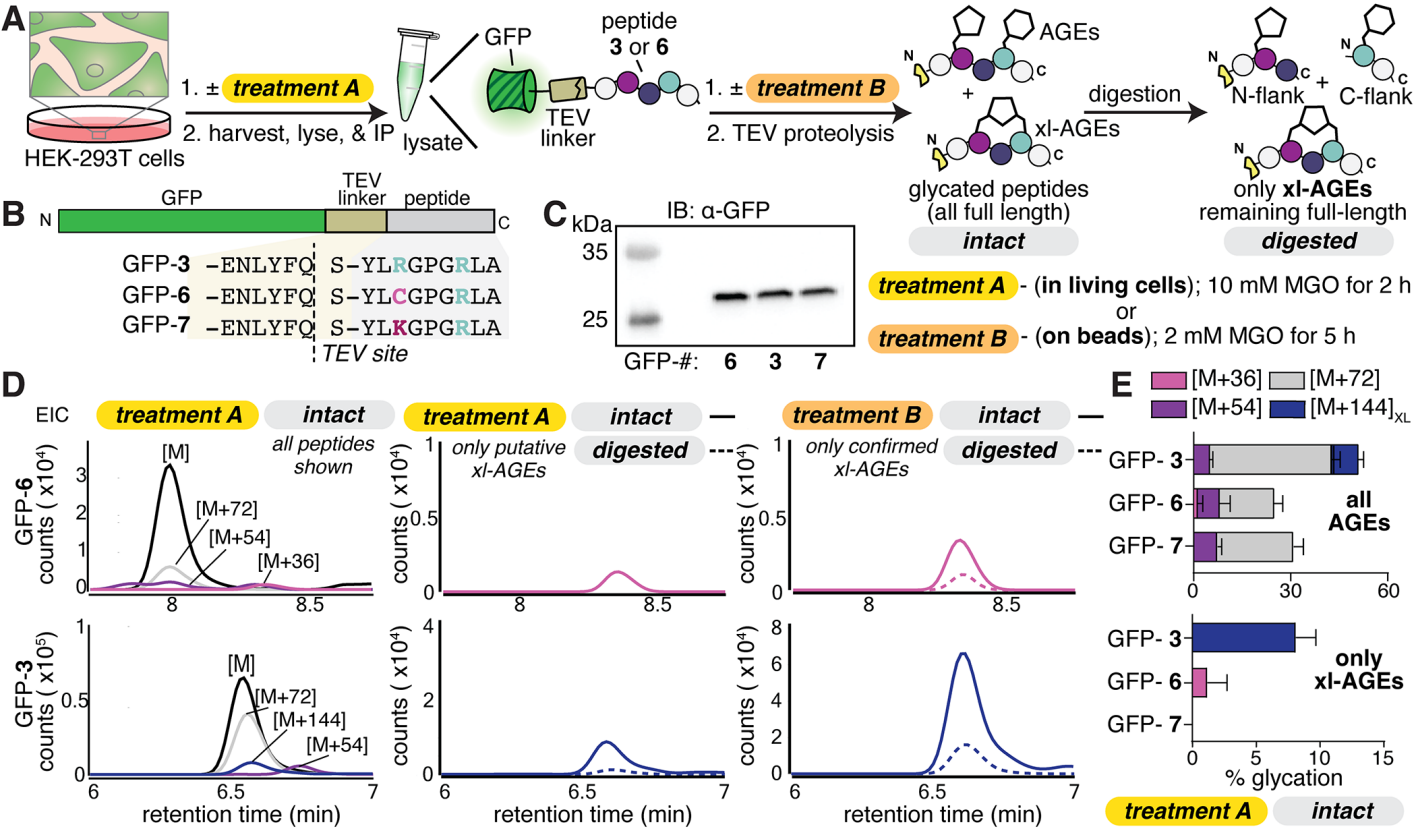
(A, B) Scheme depicting the workflow used to assess MIDAL formation in cells. Briefly, HEK-293T cells were transfected with plasmids encoding the green fluorescent protein (GFP) fused to peptide **3**, **6**, or **7**, linked via the tobacco etch virus (TEV) protease recognition site (GFP**-3**, **-6**, or **-7**, respectively). *Treatment A:* cells were treated with or without 10 mM MGO for 2 h prior to lysis. *Treatment B:* after lysis and immunoprecipitation, GFP-bound beads were treated with 2 mM MGO for 5 h. After either MGO treatment protocol, peptides were released from resin using TEV proteolysis. The resulting peptides were analyzed by LC-MS (intact) or subsequently digested with the Pro peptidase (digested). (C) Western blot analysis, probing with α-GFP antibodies, revealed that each GFP variant was expressed comparably in HEK-293T cells. (D) Representative EICs for peptides released from GFP-**6** (top row) or GFP**-3** (bottom row) after MGO treatment. *Left,* overlay of all peptide species observed in intact samples from treatment A. *Middle,* overlay of putative cross-linked species in intact (solid) and digested (dashed) samples from treatment A. *Right,* overlay of confirmed cross-linked species in intact (solid) and digested (dashed) samples from treatment B. (E) AGE distributions for intact samples obtained from MGO treatment A. Stacked bar graphs showing all AGEs (top) or only xl-AGEs (bottom) are shown as the mean ± standard deviation for each adduct. Data is from independent experiments, with *n* = 3. Legend: pink [M+36], purple [M+54], gray [M+72], blue [M+144]_XL_.

Following MGO treatment, cells were harvested, and GFP-peptide fusions were immunoprecipitated. Taking advantage of the TEV cleavage site, we then performed an on-bead TEV digestion to release the peptides of interest, which were analyzed by LC-MS. We found that GFP-**3** led to a mixture of AGEs (50.5 ± 4.3% modification), including [M+54], [M+72], [M+126], and a single peak with an [M+144] adduct (15.8 ± 3.2% modification) (Figure 5D,E). Furthermore, this [M+144] adduct was the only one remaining after treatment with the Pro peptidase. Notably, GFP-**6** also exhibited [M+54] and [M+72] AGEs, lacked [M+126] and [M+144], and instead had the [M+36] AGE, corresponding to MICA. However, when using this cross-link as a positive control, we found that we were unable to observe it after treatment with the Pro peptidase, likely due to the low levels (1.1 ± 1.5%) of MICA prior to digestion. We also noted that, consistent with our results *in vitro*, no cross-link formation was observed using GFP-**7**, though we observed [M+54] and [M+72] mono-AGEs.

To ensure that the [M+144] remaining after digestion was indeed MIDAL, we used an alternative MGO treatment protocol (*Treatment B*, Figure 5A) that would allow us to both maximize xl-AGE levels and match all cross-link retention times for MGO-treated cells, including our positive control. Specifically, in the alternative protocol, we performed on-bead MGO treatment (2 mM, 5 h), after GFP immunoprecipitation, but prior to elution with TEV protease. After this treatment, we used TEV protease to liberate the C-terminal peptides. This MGO treatment protocol led to substantially increased levels of glycation, with no change in the identities of the AGEs observed. For GFP-**3**, overall glycation levels jumped from 50.5 ± 4.3% with treatment A to 88.9 ± 4.5% for treatment B. Again, only a single peak with an [M+144] adduct was observed, but at a far greater level accounting for more than half of total glycation (56.4 ± 4.9% of total glycation). For GFP-**6**, glycation levels increased from 24.6 ± 2.9% with treatment A to 66.1 ± 1.1% for treatment B. This increase in glycation enabled us to confirm the retention time for any xl-AGEs that remained following Pro peptidase digestion. After digestion, we confirmed that the [M+36] adduct persisted, with identical retention times (Figure 5D and Table S2). This same approach was used to demonstrate that the [M+144] adduct we observed for the peptide released from GFP-**3** also persisted even after digestion, supporting its assignment as MIDAL (Figure 5D,E).

A limitation of the proof-of-concept workflow used to assess cellular MIDAL formation is that it necessitated the use of extremely high MGO concentrations that are well outside of an expected physiological range. Therefore, we performed additional experiments to explore if MIDAL could be observed under more mild treatment conditions. We explored the use of other MGO treatment conditions, including some with phosphoglycerate kinase-1 (PGK-1) or glyoxalase I (GloI) inhibitors, which have previously been used by our lab and others,^38,52–56^ but found that in many cases, we were unable to observe increases in overall glycation by Western blot (Figure S13). In other cases, conditions that increased overall glycation levels still did not produce any glycation at all, including [M+54] and [M+72] AGEs, on the peptides released after TEV proteolysis (Figure S14). However, we found that by replacing the MGO-containing media after each hour of incubation, it was possible to observe MIDAL formation on the peptide released from GFP-**3** using 2 mM MGO (Figure S14). We suspect that this approach combats the detoxification of MGO by the glyoxalase system and generates more consistent MGO levels throughout the duration of the experiment, which leads to higher levels of detectable glycation. We further note that other previously reported cross-links, MICA and MODIC, were observed only at vanishing levels or not at all, even at the highest MGO concentration used. Taken together, these results provide strong support not only that MIDAL can form in biological systems but also that it may be prevalent compared to many known AGEs, not only to other AGE cross-links.

## Discussion

Despite some recent progress,^52,57^ it remains extraordinarily challenging to monitor xl-AGE formation. Accordingly, most prior efforts to discover xl-AGEs have primarily relied on isolation (and subsequent characterization) from aged protein or tissue samples.^29,33,35^ While some xl-AGEs have been observed serendipitously,^24,25^ most recent efforts to facilitate their study have focused on the chemical synthesis of these known cross-links to generate affinity reagents.^36,58,59^ However, none of these approaches enable direct comparisons between levels of xl-AGEs and other AGEs and are poorly suited for discovering new xl-AGEs. To address this need, here we have described a peptide-based platform that enables the study of glycation cross-links. This platform allowed us not only to directly compare the prevalence of xl-AGEs to other AGEs but also to discover the first known arginine–arginine cross-link formed by methylglyoxal, which we call MIDAL. MIDAL contains a novel cross-link structure involving a surprisingly stable hemiaminal and hemiacetal. Our results suggest that MIDAL formation likely depends on the inter-Arg distance, as varying the number of intervening Gly affected the ratio of cross-links observed relative to other AGEs. We further demonstrate that MIDAL forms readily and, though its formation appears to be reversible, it also persists for days under mild, biocompatible conditions. We therefore expect that it could be an important, but so far overlooked, AGE.

Our work also shows that MIDAL can indeed form in cellular systems, further suggesting it could play a biological role that aligns more closely with cross-links that participate in functional signaling pathways^24,26^ rather than those that are considered markers of long-term damage.^20–22^ Our future work will focus on evaluating MIDAL formation on native proteins and determining its potential contribution to cellular signaling under physiologically relevant conditions. Like any PTM, xl-AGEs may alter local chemical properties and/or recruit new binding partners. However, MIDAL is also likely to impose new constraints on protein structure, dynamics, and interactions. Furthermore, our findings imply that the extent of glycation-derived cross-linking may be underestimated. For substrates with optimally positioned Arg, MIDAL can become the major AGE, surpassing even the formation of non-cross-linking AGEs on peptide substrates *in vitro*. Our future work will therefore focus on better defining the optimal inter-Arg distance to streamline the identification of authentic MIDAL substrates.

Due to the degenerate mass of MIDAL with other reported AGEs formed from MGO, including THP and double CEA (or MGH-DH) modifications, at present, it is intractable to profile MIDAL using unbiased proteomics workflows, including those designed for evaluating cross-link formation.^60,61^ We suspect that other glycation cross-links could have similarly eluded previous detection, being misattributed as another adduct with the same mass change. While we plan to develop tools and reagents that enable the detection of MIDAL substrates using proteomics, we also expect to use our peptide platform to evaluate the formation of other xl-AGEs, and their relative abundances compared to more commonly studied AGEs such as CML or MGH isomers. These studies will provide critical insights into the chemistry of glycation cross-linking events and uncover their resulting biological consequences.

## Supplementary Material



## Notes

Experimental details, material and methods, and ^1^H, ^13^C, 2D COSY, 2D HSQC NMR spectra for MIDAL (PDF)

## Abbreviations

AGE: advanced glycation end product
PTM: post-translational modifications
MGH-1: methylglyoxal-derived hydroimidazolone
MGO: methylglyoxal
MGH-DH: dihydroxyimidazolidine
CML: carboxymethyllysine
CEA: carboxyethylarginine
APY: argpyrimidine
THP: tetrahydropyrimidine
Xl: cross-linking
MODIC: methylglyoxal-derived imidazolium cross-link
MICA: mercaptomethylimidazole cross-links between cysteine and arginine
MIDAL: methylglyoxal-derived dihydroxyimidazolidine hemiacetal cross-link
TEV: tobacco etch virus
LC-MS: liquid chromatography mass spectrometry
BPC: base peak chromatogram
R.T: retention time
PBS: phosphate-buffered saline
EIC: extracted ion chromatogram
GO: glyoxal
D4-MGO: deuterated methylglyoxal
GFP: green fluorescent protein
IP: immunoprecipitation
PGK-1: phosphoglycerate kinase-1
GloI: glyoxalase I
